# Alzheimer’s disease prediction via an explainable CNN using genetic algorithm and SHAP values

**DOI:** 10.1371/journal.pone.0337800

**Published:** 2026-01-16

**Authors:** Mohammad Zahedipour, Mohammad Saniee Abadeh, Shakila Shojaei

**Affiliations:** 1 Faculty of Electrical and Computer Engineering, Tarbiat Modares University, Tehran, Iran; 2 School of Computer Science, Institute for Research in Fundamental Sciences (IPM), Tehran, Iran; 3 University of Groningen, Groningen, The Netherlands; Jaypee University of Information Technology, INDIA

## Abstract

Convolutional neural networks (CNNs) are widely recognized for their high precision in image classification. Nevertheless, the lack of transparency in these black-box models raises concerns in sensitive domains such as healthcare, where understanding the knowledge acquired to derive outcomes can be challenging. To address this concern, several strategies within the field of explainable AI (XAI) have been developed to enhance model interpretability. This study introduces a novel XAI technique, GASHAP, which integrates a genetic algorithm (GA) with SHapley Additive exPlanations (SHAP) to improve the explainability of our 3D convolutional neural network (3D-CNN) model. The model is designed to classify magnetic resonance imaging (MRI) brain scans of individuals with Alzheimer’s disease and cognitively normal controls. Deep SHAP, a widely used XAI technique, facilitates the understanding of the influence exerted by various voxels on the final classification outcome (Lundberg SM, Lee SI. A unified approach to interpreting model predictions. In: Advances in Neural Information Processing Systems, 2017. 4765–74. https://doi.org/10.5555/3295222.3295230). However, voxel-level representation alone lacks interpretive clarity. Therefore, the objective of this study is to provide findings at the level of anatomically defined brain regions. Critical regions are identified by leveraging their SHAP values, followed by the application of a genetic algorithm to generate a definitive mask highlighting the most significant regions for Alzheimer’s disease diagnosis (Shahamat H, Saniee Abadeh M. Brain MRI analysis using a deep learning based evolutionary approach. Neural Netw. 2020;126:218–34. https://doi.org/10.1016/j.neunet.2020.03.017 PMID: 32259762). The research commenced by implementing a 3D-CNN for MRI image classification. Subsequently, the GASHAP technique was applied to enhance model transparency. The final result is a brain mask that delineates the pertinent regions crucial for Alzheimer’s disease diagnosis. Finally, a comparative analysis is conducted between our findings and those of previous studies.

## 1. Introduction

The field of medical image analysis has been significantly advanced by developments in convolutional neural networks (CNNs). Researchers increasingly favor these networks due to their capacity to obviate the need for manual feature engineering [3]. The utility of deep learning models has been demonstrated in various tasks, including object identification [4–6], segmentation [7,8], and classification [3,9,10]. Nevertheless, the lack of explainability in these opaque models has raised concerns regarding their application in critical domains such as medicine. To address this issue, scholars have developed XAI methodologies aimed at improving model transparency. These methodologies can be broadly categorized into three groups: visual techniques [11,12], textual techniques [13,14], and example-based techniques [15–17]. This paper presents a novel methodology to enhance the interpretability of CNN models in medical image analysis. The proposed strategy employs Deep SHAP, an approach developed by Lundberg and Lee [1], which integrates Shapley values with the DeepLift algorithm, as described by Shrikumar et al. [18], in a post hoc and model-specific manner. The methodology used in this study comprises five sequential steps. First, brain MRI images were preprocessed. Next, a CNN was trained using MRI scans from Alzheimer’s patients and cognitively normal individuals derived from the Alzheimer’s Disease Neuroimaging Initiative (ADNI) dataset [19]. SHAP values were then computed to improve model explainability. However, SHAP values provide outputs at the voxel level. To facilitate comparison with existing research and offer actionable results to medical practitioners, it is necessary to report findings in terms of brain regions. Moreover, the SHAP method is inherently localized and does not yield a definitive brain area mask. To overcome these limitations, we incorporated a genetic algorithm—previously described in our work [2]—into our methodology. The initial phase involved generating a population using heatmaps derived from SHAP values. Brain area masks were then applied to segment each heatmap, and a score for each region was calculated using the SHAP values and a prescribed formula. A numerical value from a predefined set S = {1, 2, 3, 4} was subsequently assigned to each region. The following steps of the genetic algorithm were then defined. Ultimately, the performance of the proposed GASHAP framework was evaluated by assessing the accuracy of the generated brain areas and the convergence properties of the genetic algorithm.

## 2. Related works

We have classified related works into the following areas: Alzheimer’s classification and explainability methods for neural networks.

### 2.1. Alzheimer’s classification

Numerous studies have presented diverse neural network architectures for the purpose of classifying medical images, as this task holds significant benefits for the development of XAI techniques. Consequently, numerous studies have employed image classification techniques for the purpose of diagnosing Alzheimer’s disease [20–22]. In recent years, transfer learning has gained prominence due to its capacity to optimize time and resources by obviating the need to train several machine learning models from the ground up in order to do similar tasks. One example involves the proposal of a technique that employs deep neural convolutional networks, transfer learning, MRI inside the Visual Geometry Group (VGG) 16 and VGG 19 models [23]. This study additionally demonstrated that Alzheimer’s disease (AD) may be classified into four distinct groups, which are commonly recognized by medical professionals, through the use of computer algorithms. The authors of the study [24] present a deep feature-based methodology for the identification of Alzheimer’s disease stages. In order to accomplish this task, the initial layers of a pre-existing AlexNet model are utilized, and afterwards, the deep features are learned using a convolutional neural network. In this study, the deep features that have been extracted are subjected to classification using several machine learning approaches, such as K-nearest neighbor (KNN), random forest (RF), and support vector machine (SVM). Ebrahimi-Ghahnavieh et al. [25] present a strategy that combines CNN and recurrent neural networks (RNN) with the aim of improving classification performance. The initial step involves the partitioning of every three-dimensional MRI image into separate two-dimensional images. Subsequently, each of these two-dimensional images undergoes a distinct retraining process utilizing a distinct two-dimensional CNN. In order to build a significant correlation between these sequences and facilitate the process of decision-making, the Long Short-Term Memory (LSTM) model is employed to aggregate these inputs. The limited availability of comprehensive medical imaging training data is a challenge to the performance of CNNs and has the potential to result in overfitting. The study conducted by Acharya et al. [26] intended to employ transfer learning models, including VGG16, ResNet-50, and AlexNet, along with convolutional neural networks, to classify MRI scans of patients with Alzheimer’s disease into various classifications. Furthermore, this approach successfully classified several stages of Alzheimer’s disease, including mild cognitive impairment, mild Alzheimer’s disease, moderate Alzheimer’s disease, and severe impairment, with a high accuracy rate of 95.70%. Recent studies have further expanded the scope of Alzheimer’s Disease (AD) classification by incorporating explainability and diverse data types. For instance, a study utilizing a large clinical dataset from the National Alzheimer’s Coordinating Center employed traditional machine learning models such as SVM and RF with explainability techniques like SIRUS, CAR, SHAP, and LIME, achieving high accuracies (up to 99% for binary classification) [27]. Another study proposed a multilayer multimodal detection and prediction model using MRI, PET, and genetic data with RF classifiers, achieving accuracies of 94.4% for AD detection and 86.8% for progression prediction, enhanced by SHAP and Decision Trees for explainability [28]. Additionally, a deep transfer learning approach using ensemble models (e.g., VGG16, DenseNet) on MRI data achieved 96% accuracy, employing XAI techniques like saliency maps and GRAD-CAM, providing a direct comparison to GASHAP’s performance [29]. Recent advancements include multimodal deep learning with MRI, PET, and genetic data, achieving high diagnostic accuracy [30,31], and graph neural networks for connectivity-based AD classification [32]. Transfer learning with VGG19 and DenseNet has been explored, with SHAP and LIME enhancing interpretability [33], while random forest models predict (Mild Cognitive Impairment (MCI) to Alzheimer’s Disease (AD) progression (MCI-to-AD)) transition using multimodal data [34]. Three-class classification (control, MCI, AD) using deep learning and SHAP further refines diagnostic granularity [35].

Convolutional Neural Networks (CNNs) have demonstrated remarkable capability in automatically learning spatial and hierarchical features directly from medical images, eliminating the need for handcrafted feature extraction that traditional machine learning algorithms such as Support Vector Machines (SVM), Random Forests (RF), and k-Nearest Neighbors (k-NN) typically require. This ability allows CNNs to capture subtle structural and textural patterns within MRI data that may not be detectable through conventional techniques. Furthermore, CNNs provide scalability to high-dimensional input data, enabling end-to-end optimization for disease classification tasks such as Alzheimer’s diagnosis.

Despite these advantages, CNN-based approaches also present certain limitations. They often demand substantial computational resources and large annotated datasets for effective training, which can be restrictive in medical imaging domains where data acquisition is expensive and labeling is time-consuming. Additionally, CNNs have been criticized for their limited interpretability, as the internal decision-making process is not easily understandable to clinicians. This “black-box” nature underscores the necessity for XAI frameworks such as GASHAP, which aim to enhance model transparency while maintaining diagnostic accuracy.

A comparative summary highlighting the relative strengths and weaknesses of CNNs versus conventional machine learning methods is provided in Table 8, demonstrating how deep architectures improve representational power at the cost of increased complexity and reduced interpretability

### 2.2. XAI methods

A recent systematic review of XAI in Alzheimer’s disease classification analyzed 37 studies, covering frameworks such as LIME, SHAP, GradCAM, and Layer-wise Relevance Propagation (LRP) [36]. It highlighted critical gaps, including the need for validation with medical professionals and comprehensive datasets, which the GASHAP method addresses through its focus on MRI and precise brain region identification. In recent times, a considerable amount of scholarly attention has been directed toward the development of strategies aimed at enhancing the transparency of neural network models. This has been achieved through the integration of many algorithms or the independent use of diverse methodologies. LRP, initially proposed by Bach et al. [37], is an algorithm that is widely employed and considered to be highly intriguing. The utilization of LRP for the visualization of the CNN model, as described by Böhle et al. [38], is employed in the context of Alzheimer’s disease (AD) classification using MRI. In order to highlight the benefits of the LRP algorithm, a comparison is being made between this algorithm and the guided backpropagation (GB) technique, which is a gradient-based method. Ultimately, the researchers revealed significant cerebral regions that are crucial for the diagnosis of Alzheimer’s disease. The output of a CNN model can be visualized as a heatmap by employing Shapley additive explanations (SHAP), a technique that bears resemblance to LRP (Layer-wise Relevance Propagation) as proposed by Shapley [39]. In the study conducted by van der Velden et al. [40], a 3-dimensional regression CNN was utilized for the purpose of evaluating volumetric breast density. The primary benefit of the methodology suggested in this research article lies in its ability to circumvent the expensive process of conducting 3-dimensional voxel-level segmentations of the breast and fibroglandular tissue. One notable feature of the methodology outlined in this study is the use of a density score. In cases where radiologists encounter uncertainty regarding the density score, they may turn to the Shapley additive explanations for guidance. Seo et al. [41] introduced a regional, multi-scale, model-agnostic approach aimed at enhancing the interpretability of deep neural networks through the use of visualization techniques. This approach utilizes a region-based methodology to estimate the individual contribution of each feature towards the ultimate outcome. This estimation is achieved by omitting a specific region, which is generated through the sampling of a normal distribution formed based on the boundary prior. This methodology involves the segmentation of images into distinct layers, thereby facilitating a more precise and comprehensive elucidation of the overall shape. The degree of detail in the explanation is enhanced through the process of segmenting the photos into higher tiers. The study conducted by Sudar et al. [42] focuses on the classification of MRI Alzheimer’s brain images using a CNN architecture called VGG-16. The experiment uses the LRP technique to interpret the features utilized by the model for prediction. The collection has around 6400 photographs that have been categorized into four separate classifications. The results of this study suggest that the application of the VGG-16 model and the LRP approach is effective in the classification of images associated with Alzheimer’s disease. Furthermore, these findings provide significant insights into the unique attributes that are employed for precise forecasting. The genetic algorithm is a metaheuristic algorithm that operates on a population and can be employed for the interpretation of deep neural networks. In Shahamat and Abadeh [2], we focused on the identification of significant brain areas associated with autism and Alzheimer’s disease through the use of a genetic algorithm. Initially, a CNN is trained. Subsequently, genetic algorithm properties, including population initialization, fitness evaluation, crossover, and mutation, were established. The chromosome encoding utilized in this approach is derived from the Harvard brain atlas, where each gene within a chromosome is assigned a random value from the set S = {0, 1, 2, 3}. The brain regions that have been acquired are identified as crucial brain regions in the diagnosis of Alzheimer’s disease and autism. In Shojaei et al. [43], which is another important work in the field of XAI, we presented a research inquiry that explores the utilization of deep neural networks (DNNs) and CNNs for the purpose of diagnosing Alzheimer’s disease (AD) by analyzing MRI data. The integration of a genetic algorithm-based occlusion map technique with backpropagation-based explainability approaches was employed to identify the specific brain regions that significantly contribute to the output of the network. The extracted regions underwent a comparative examination with prior studies, which revealed their effectiveness in diagnosing Alzheimer’s disease. This conclusion was supported by professionals in the field of Alzheimer’s research. The model achieved an accuracy of 87% during the 5-fold cross-validation procedure, indicating a performance level comparable to that observed in prior research in a similar context. In addition, we achieved a validation accuracy of 93% using only 29 brain regions and implementing the lrp_z_plus_fast explainability approach. This study demonstrates the efficacy of a merged approach for the diagnosis of Alzheimer’s disease (AD) and contributes to the existing body of literature in this field. Ahn et al. [44] developed a genetic algorithm with the purpose of extracting meaningful features from the complete dataset. The researchers in this study provided a traffic classifier based on ResNet and afterwards discussed the importance of each feature by establishing the dominance rate. The proposed methodology employs a genetic algorithm to generate a feature selection mask. The classifier assesses the performance of each mask and selects those with the most favorable fitting scores. Shad et al. [45] focused on using the LIME framework for XAI in Alzheimer’s disease (AD) diagnosis. They aimed to improve the interpretability of deep learning models by providing explanations and visualizations using the LIME framework. Their hybrid dataset, which included MRI imaging data, allowed them to classify patients into different stages of dementia and distinguish AD patients from normal individuals. The researchers highlighted the importance of transparency in the medical field and demonstrated the potential of the LIME-based XAI framework in enhancing AD diagnosis by aiding in early detection and treatment planning. Bordin et al. [46] addressed XAI in Alzheimer’s disease (AD) classification using deep learning models. They trained a DL model on MRI scans of individuals with AD and healthy controls, achieving good performance. The occlusion sensitivity method was used to understand the DL model’s decision-making process, revealing the importance of white matter hyperintensity (WMH) lesions in AD identification. DL models can effectively utilize clinical information, particularly WMHs, as a biomarker for AD dementia. XAI methods provide insights and enhance trust in DL approaches in clinical settings. Recent advancements in XAI for AD classification emphasize SHAP for interpreting deep learning models, with studies using SHAP to identify key MRI-based biomarkers [35,47–51] and multimodal features like cognitive scores and genetic data [30,31,34,52,53]. Systematic reviews highlight the role of SHAP and LIME in AD detection, addressing clinical validation needs [54]. SHAP has been applied to graph-based models for brain connectivity analysis [55] and multitask deep learning for MCI-to-AD progression [56]. Alternative XAI methods, such as Grad-CAM and LIME, provide visual and local explanations for 3D-CNNs and transfer learning models [33,57,58], while LRP identifies critical brain regions [59]. Integrated SHAP-LIME frameworks offer hybrid interpretability [60], enhancing diagnostic transparency. The GASHAP method, combining genetic algorithms with SHAP, aligns with these trends, particularly in SHAP-driven region-based explainability [51].

## 3. Materials and methods

**Fig 1:**
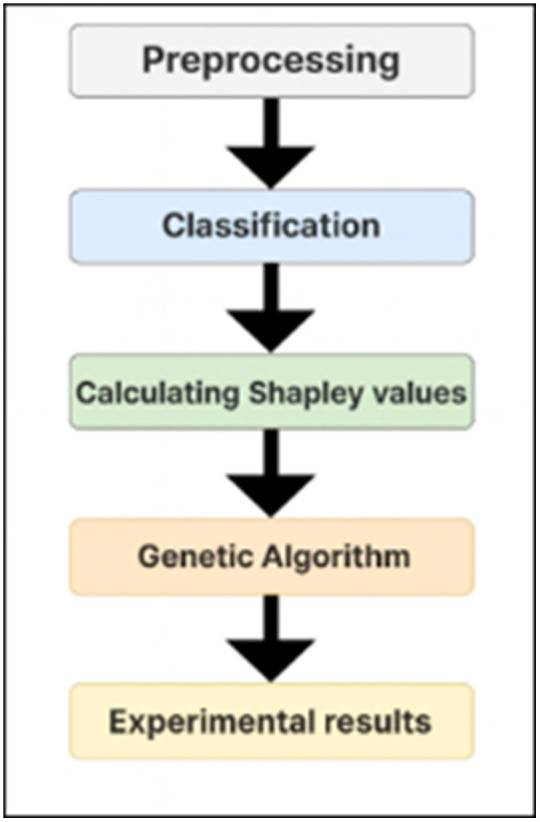
Overall workflow of GASHAP.

The *GASHAP* methodology encompasses five critical stages: data preprocessing, classification, calculation of Shapley values, genetic algorithm implementation, and experimental results. The overall procedure of the *GASHAP* methodology is depicted in Fig 1.

### 3.1. Ethical considerations

This study utilized de-identified MRI data from the Alzheimer’s Disease Neuroimaging Initiative (ADNI), with approval from the ADNI Data and Publications Committee and relevant institutional review boards [19].

### 3.2. Data preprocessing

The MRI data used in this study were obtained from the ADNI repository (https://adni.loni.usc.edu/). Following the protocol proposed by Shahamat and Saniee Abadeh [2], a total of 140 T1-weighted MRI scans were selected, consisting of 70 scans from patients diagnosed with Alzheimer’s disease (AD) and 70 scans from cognitively normal (CN) controls. The demographic variables (age and gender) were approximately balanced across the two groups to minimize sampling bias. All MRI volumes were preprocessed using the FSL toolbox. Each image was reoriented to the standard MNI152_T1_2 mm space, skull-stripped, and intensity-normalized. Subsequently, the images were cropped to an 80 × 80 × 80 voxel subvolume centered on the brain to remove redundant background regions and reduce computational cost. The dataset was divided into training, validation, and testing subsets with a 70–15–15% ratio, maintaining class balance across subsets. In addition, a 5-fold cross-validation procedure was implemented to ensure the robustness and generalization of the model. Each MRI volume was normalized to zero mean and unit variance before being fed into the CNN model. **Implementation details**: All experiments were implemented in Python using the TensorFlow deep learning framework within the Google Colab environment. Training was performed on a GPU provided by Colab. Random seeds were fixed to ensure reproducibility. All analyses and training sessions were conducted under a consistent software setup.

### 3.3. CNN classification

The CNN architecture used in this study followed the design proposed by Shahamat and Saniee Abadeh [2]. The model consisted of three 3D convolutional blocks, each followed by a rectified linear unit (ReLU) activation and a 2 × 2 × 2 max-pooling layer. The first, second, and third convolutional layers employed kernel sizes of 5 × 5 × 5, 3 × 3 × 3, and 3 × 3 × 3, respectively. The output of the final convolutional block was flattened and connected to two fully connected layers containing 1024 and 2 neurons, respectively. A softmax activation function was applied in the output layer to produce class probabilities for Alzheimer’s disease (AD) and cognitively normal (CN) subjects.

The network was trained from scratch using the **Adadelta** optimizer with an initial learning rate of **0.05** and a decay rate of **0.95**. The **categorical cross-entropy** loss function was used to measure prediction error. The model was trained with a batch size of **32** for up to **30,000 iterations** (approximately 100 epochs). An **early stopping** criterion was applied when the validation loss did not improve for 10 consecutive epochs.

The model achieving the highest validation accuracy was saved for further analysis. This trained CNN was subsequently used to compute voxel-level SHAP values, which served as input to the genetic algorithm phase for generating interpretable regional masks (Table 1).

**Table 1:** The 3D-CNN architecture.

Layer Type	Kernel Size	# Filters	Scan Size	Activation	Other Details
Input	—	—	80 × 80 × 80 × 1	—	Input volume of size 80x80x80 and 1 channel
Dropout	—	—	80 × 80 × 80 × 1	—	Dropout layer with 50% keep probability
Convolution	5 × 5 × 5	8	80 × 80 × 80 × 8	ReLU	Convolutional layer with 5x5x5 kernel, 8 filters, and ReLU activation
Max Pooling	2 × 2 × 2	—	40 × 40 × 40 × 8	—	Max pooling layer with 2x2x2 pool size
Convolution	3 × 3 × 3	16	40 × 40 × 40 × 16	ReLU	Convolutional layer with 3x3x3 kernel, 16 filters, and ReLU activation
Max Pooling	2 × 2 × 2	—	20 × 20 × 20 × 16	—	Max pooling layer with 2x2x2 pool size
Convolution	3 × 3 × 3	32	20 × 20 × 20 × 32	ReLU	Convolutional layer with 3x3x3 kernel, 32 filters, and ReLU activation
Max Pooling	2 × 2 × 2	—	10 × 10 × 10 × 32	—	Max pooling layer with 2x2x2 pool size
Flattening	—	—	1 × 32000	—	Flattening layer to convert the volume into a 1-dimensional vector
Fully Connected	32000x1024	—	1 × 1024	ReLU	Fully connected layer with 32000x1024 weights and ReLU activation
Dropout	—	—	1 × 1024	—	Dropout layer with 50% keep probability
Fully Connected	1024x2	—	1 × 2	—	Fully connected layer with 1024x2 weights
Softmax Layer	—	—	1 × 2	Softmax	Softmax activation layer for classification
Classification Layer	—	—	Patient vs. Normal	—	Final classification layer for distinguishing between patient and normal samples

### 3.4. Calculating shapley values

The SHAP methodology is a game-theoretic strategy that provides a concise approach for interpreting machine learning models, especially those with a black-box nature [1]. The SHAP framework offers implementations that can be applied a variety of machine learning models, such as Kernel SHAP, Tree SHAP, and Deep SHAP. The SHAP approach produces a unique and well-defined solution that exhibits three desirable characteristics: local accuracy, missingness, and consistency.

**Property 1** (local accuracy)

When the explanation model agrees with the original model, The local accuracy equation is shown in Equation 1.

**Equation 1.** Local Accuracy:


$$f(x)=g\left(x^{\prime }\right)=\varphi_{\text{0}\;}+\sum_{i=\text{1}}^{M}\varphi_{i\;}{x^{\prime }}_{i}$$ (1)

**Property 2** (Missingness)

Features that ${x^{\prime }}_{i}=\text{0}$ are constrained by missingness have no attributable influence. This desirable quality is displayed by Equation 2.

**Equation 2.** Missingness:


$${x^{\prime }}_{i}=\text{0}\;\to \varphi_{i}=\text{0}$$ (2)

**Property 3** (Consistency)

Equation 3 explains the consistency property. Let $f_{x}\left(z^{\prime }\right)=f_{x}\left(h_{x}(z^{\prime })\right)$ and $z^{\prime }\backslash i$ denote setting ${z^{\prime }}_{i}=\text{0}$. For any two models f and $f^{\prime },$ if:

**Equation 3.** Consistency:


$${f^{\prime }}_{x}\left(z^{\prime }\right)-{f^{\prime }}_{x}\left(z^{{}^{\prime }\backslash }i\right)\geq f_{x}\left(z^{\prime }\right)-f_{x}(z^{{}^{\prime }\backslash }i)$$ (3)

for all inputs $z^{\prime }\in {\left\{\text{0},\text{1}\right\}}^{M}$, then $\varphi_{i}(f^{\prime },x)\geq \varphi_{i}(f,x)$.

**Fig 2:**
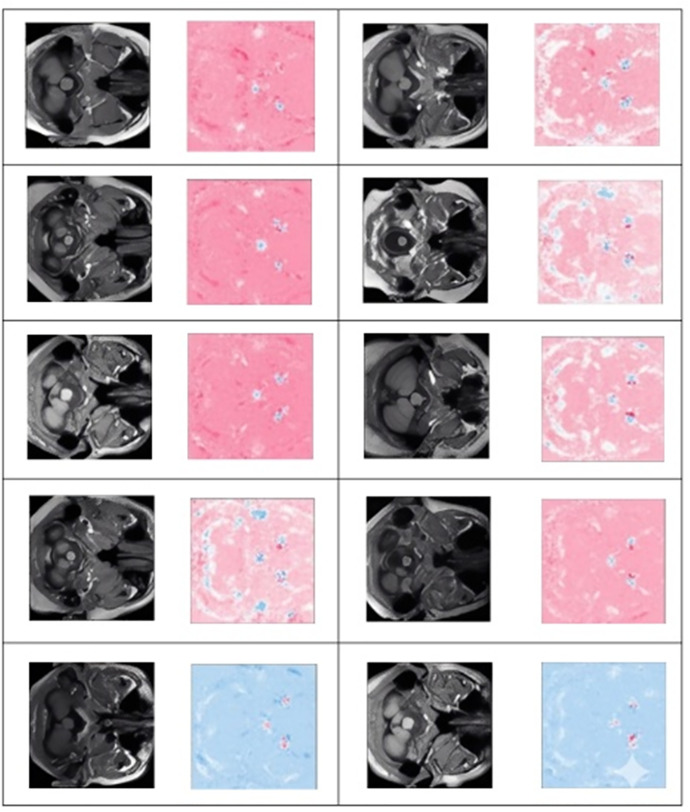
10 samples of MRI images and their corresponding heatmaps.

Deep SHAP is a viable approach for elucidating the workings of deep neural network models. In this study, we employed a novel approach to increase transparency in black-box models by identifying crucial voxels in brain MRI images. This step of the GASHAP process results in the generation of heatmaps corresponding to each MRI image, as depicted in Fig 2. In the subsequent section, we describe the use of Shapley values in the generation of chromosomes for the genetic algorithm.

### 3.5. Genetic algorithm

In the previous stage, the objective was to identify the voxels within the MRI images that exerted the most significant influence on the final outcome. To facilitate interpretation, the Harvard-Oxford cortical and subcortical structural atlas was used to delineate a total of 96 distinct brain regions, as shown in Table 2. This approach enabled analysis of results at the brain region level. Masks were generated using the previously computed Shapley values, as depicted in Fig 7. However, it is important to note that the SHAP technique, which provides explanations at a local level, may not yield precise outcomes when averaging Shapley values across all MRI scans. To address this limitation, a technique was utilized to convert each heatmap into a chromosome. The implemented genetic algorithm consists of five distinct steps, outlined below:

**Table 2:** Predefined names of brain regions and their corresponding IDs.

ID	Name
1	Left Angular Gyrus
…	…
13	Left Inferior Temporal Gyrus, anterior division
14	Left Inferior Temporal Gyrus, posterior division
…	…
62	Right Inferior Temporal Gyrus, posterior division
63	Right Inferior Temporal Gyrus, temporooccipital part
64	Right Insular Cortext
65	Right Intracalcarine Cortex
…	…
89	Right Superior Temporal Gyrus, posterior division
90	Right Supracalcarine Cortex
…	…
96	Right Temporal Pole

1 Initial population
2 Fitness function
3 Selection method
4 Crossover
5 Mutation

This section will outline the stages involved in the implementation of the genetic algorithm. The fitness function, crossover, selection strategy, and mutation employed in this study closely adhere to the approach described in our previous study [2]. However, we have made modifications to the initialization of the population based on the heatmaps generated by Deep SHAP.

#### 3.5.1. Initial population.

3.5.1.1. *Chromosome Encoding*: Based on the findings presented in the Harvard-Oxford Brain Region Atlas (Table 2), it was observed that there were a total of 96 distinct brain regions. Consequently, in our study, we designated each chromosome as a vector with a length of 96. The value assigned to each element of the vector is determined based on the corresponding values derived from the significance of every value within set S is provided in Table 3.

**Table 3:** Brain region importance.

Gene value	Meaning
3	Very Very Important Region(VVIR)
2	Very Important Region(VIR)
1	Important Region(IR)
0	Unimportant Region)UR)

**Fig 3:**
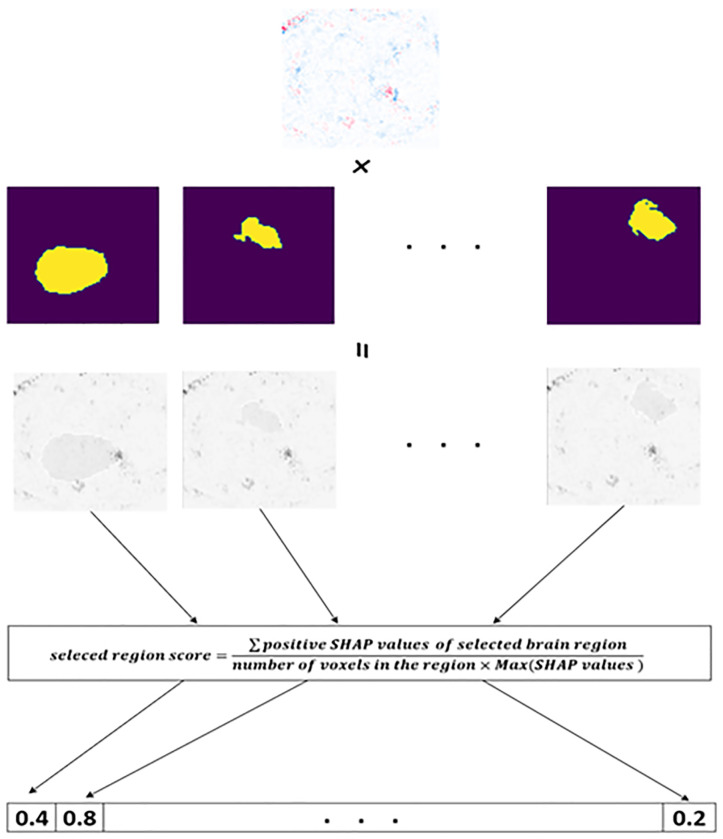
The process of score calculation for a heatmap.

3.4.1.2. *Score calculation*: For each heatmap, the brain regions described in the Harvard-Oxford Atlas must be used to assign values from Table 3 to the genes on each chromosome. Subsequently, a score is calculated for each region by employing the mathematical formula presented in Equation 4. The diagram in Fig 3 illustrates the overall process of score computation. It should be noted that Max(SHAP values ) represents the highest value among the SHAP values inside the designated region.

**Equation 4.** Score Calculation Formula Based on SHAP Values:


$$seleced\;region\;score=\frac{\sum positive\;SHAP\;values\;of\;selected\;brain\;region}{number\;of\;voxels\;in\;the\;region\times Max(SHAP\;values\;)}$$ (4)

**Fig 4:**
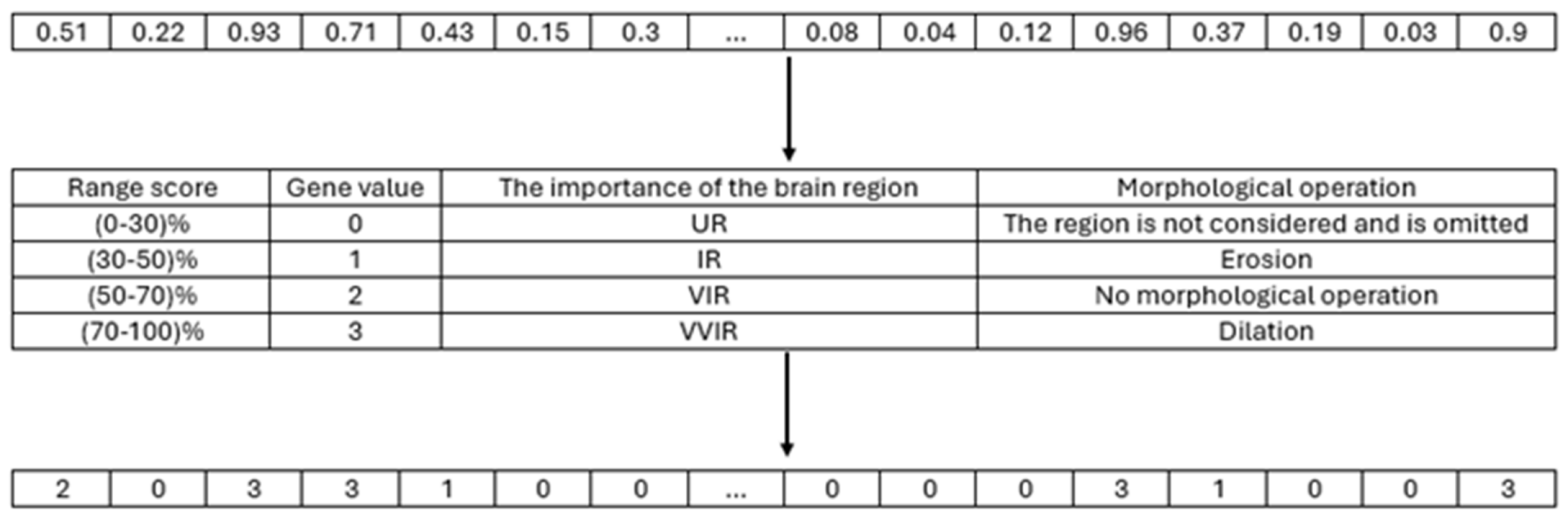
The Process of Applying Ranges to the Score Array for Creating Chromosomes. 1. After calculating the SHAP values and applying Equation 4, a score is assigned to each region in every MRI image. Consequently, each heatmap contains an array in which the scores of each region are stored. 2. Each area is placed in a range according to its score, and a value from the score range table is assigned to it. 3. That is, the first 30 percent that have the lowest score are assigned a value of 0, those that are between 30 and 50 percent are assigned a value of 1, the areas that are between 50 and 70 are assigned a value of 2, and the rest are assigned a value of 3.

**Fig 5:**
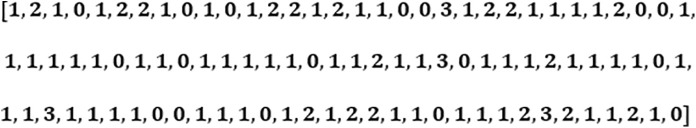
An example of a generated chromosome with 96 genes.

**Fig 6:**
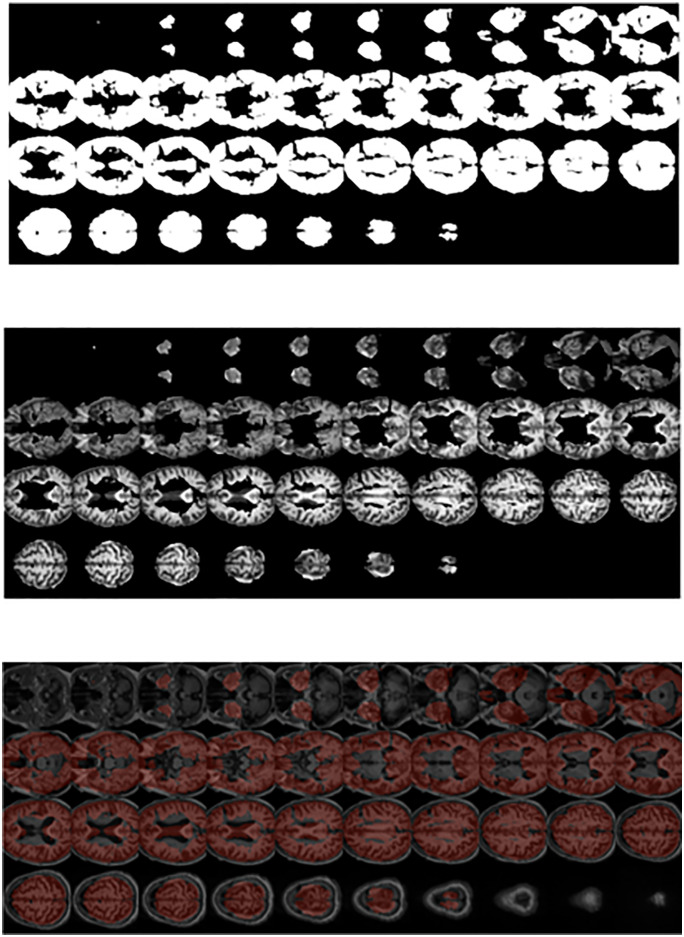
The corresponding regions of the chromosome produced in Fig 5.

**Fig 7:**
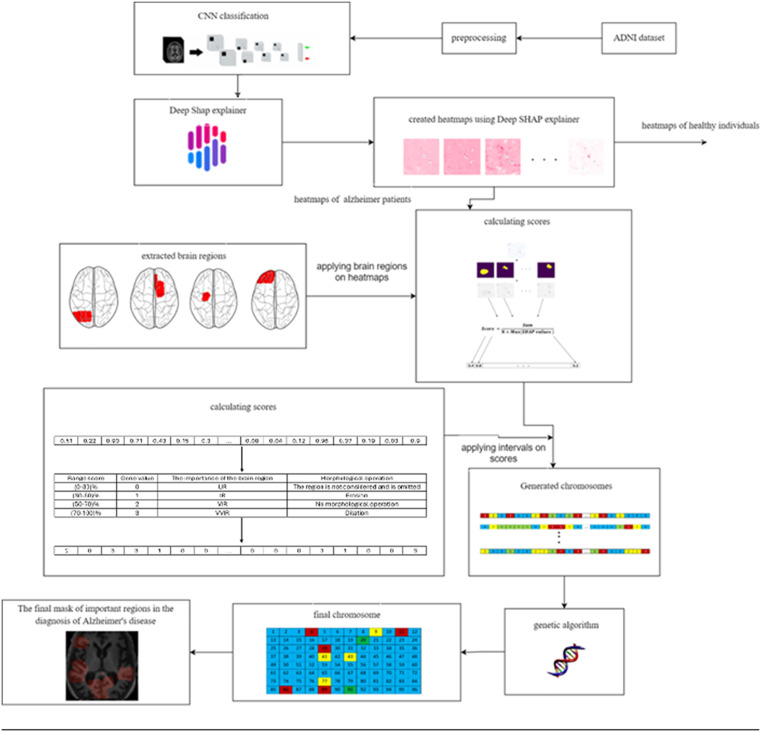
Overall diagram of chromosome-generating flow.

In the course of the investigation, it becomes clear that a location with a higher score is indicative of a higher level of criticality. In order to preserve the accuracy of the model, the equation eliminated zeros and negative SHAP values. The numerical value assigned to each region, as indicated by Equation 4, spans the range of zero to one. Following this, a numerical value from the set S = {0, 1, 2, 3} was allocated to each brain region, taking into account the outcomes of the preceding computation. The resulting brain masks are illustrated in Fig 4. The scores were arranged in ascending order, and a value of 0 was assigned to the lowest 30% of the group based on their scores. In a similar manner, numerical values of 1 were allocated to scores falling within the range of 30–50%, values of 2 were assigned to scores falling within the range of 50–70%, and values of 3 were assigned to scores falling within the range of 70–100%. The brain region exhibiting a gene value of 0 was omitted during the generation of the brain mask. In the event that a gene possessed a value of 1, morphological erosion was employed to diminish the dimensions of the associated brain region, given its lower score and potential scarcity of crucial voxels. Conversely, a gene exhibiting a value of 3, which is assigned a higher score according to SHAP, may possess a greater number of valuable voxels. Consequently, the associated region was expanded through the use of morphological dilation. Fig 5 illustrates the developed chromosome, while Fig 6 depicts the transformation outcome of the generated chromosome into a brain mask. The schematic representation of the chromosomal generation process in GASHAP is depicted in Fig 7.

#### 3.5.2. Fitness function.

As stated previously, each chromosome represents a distinct manifestation of the brain mask. The fitness function is defined according to Equation 5 to evaluate the efficacy of the generated chromosomes. The evaluation of the precision of each scan involves an initial conversion of each chromosome into a brain mask, which is subsequently used to process all input MRI pictures. The fitness value is subsequently computed by taking the reciprocal of the quantity representing the count of pertinent brain regions and combining it with a weighted mean of the model’s precision. The equation presented in question 5 provides a comprehensive definition of the fitness function: it represents the accuracy of processed input scans, the inverse of the non-zero elements, and variables that optimize both accuracy and the number of selected brain regions.

**Equation 5**. Fitness function:


$$fitnessValue=\alpha f_{\text{1}}+\beta f_{\text{2}}$$ (5)

**Equation 6**. Mask accuracy where TP, FP, TN, and FN denote true positives, false positives, true negatives, and false negatives:


$$f_{\text{1}}=\frac{TP+TN}{TP+TN+FP+FN}$$ (6)

**Equation 7**. Inverse number of selected brain regions:


$$f_{\text{2}}=\frac{\text{1}}{\sum_{i=\text{1}}^{\text{9}\text{6}}{CH}_{i}>\text{0}}$$ (7)

The GA was employed to optimize the SHAP-based regional masks following the procedure proposed in our previous work [2]. Each chromosome encoded a binary vector representing candidate brain regions, and the fitness function was computed as a weighted combination of classification accuracy and mask compactness.

The GA was configured with a **population size of 200**, a **maximum of 500 generations**, a **crossover probability of 0.4**, and a **mutation probability of 0.6**. Tournament selection was used to choose parents for reproduction. The evolution was terminated either when the maximum number of generations was reached or when the improvement in mean fitness between consecutive generations fell below 0.001.

The weighting coefficients **α = 0.025** and **β = 0.975** in the fitness function were adopted from Shahamat and Saniee Abadeh [2], where they were shown to provide a good trade-off between accuracy and interpretability. The small α value gives a minor penalty to mask size to prevent excessive pruning of relevant voxels, while the large β value emphasizes classification accuracy to maintain diagnostic reliability. Preliminary experiments in this study confirmed that these parameter values produced stable convergence behavior and high validation accuracy.

#### 3.5.3. Selection method.

The parental candidates for the subsequent generation are chosen from the population as the most appropriate answers. Various selection strategies can be employed to accomplish this task. In the present study, the roulette wheel method has been employed. Equation 8 can be used to compute the likelihood associated with the selection of each individual member.

**Equation 8**. The probability of selecting ith member of population:


$$Prob(i)=\frac{fitnessValue(i)}{\sum_{j=\text{1}}^{n}fitnessValue(j)}$$ (8)

#### 3.5.4. Crossover.

Two parents, *A* and B, are selected, then a random number *n* between 1 and 96 is chosen, and a new offspring is generated with the first *n* genes of *A* and the remaining genes of *B*.

#### 3.5.5. Mutation.

In this step, a random gene is chosen, and a random value is assigned to all selected individuals.

## 4. Experimental results

In this study, we used a CNN and the previously mentioned architectural elements to categorize MRI scans of the Alzheimer’s disease (AD)-affected brain. Nevertheless, the model’s fundamental learning mechanism remained incompletely understood, raising doubts about its trustworthiness. To address these issues, SHAP values were calculated using the DeepExplainer library. To facilitate the identification of important voxels in the MRI images and make the model easier to understand. In the subsequent part, we enhanced the clarity of our findings by utilizing the Harvard-Oxford Atlas of the Brain and articulating the outcomes in relation to specific brain regions. The process involved mapping brain regions to individual heatmaps, then calculating scores for each region using a specific equation. The primary aim of this research is to generate a comprehensive map of brain regions that are indicative of critical areas affected by Alzheimer’s disease. One possible method for determining the value of each voxel in the final mask is to compute the average. Nevertheless, it is important to acknowledge that this approach may yield inaccurate results due to outliers in the dataset. In our previous work [2], we introduced a technique, GAMB, to determine the ultimate mask. This involves applying a genetic algorithm to compute the final mask. The GAMB chromosomes contain 96 genes, each assigned a random value from 0 to 3. These values then serve as the driving force behind morphological procedures. The initial population of the genetic algorithm is large and consists of individuals selected at random. The process of initializing chromosomes in GASHAP offers three significant advantages in the establishment of the initial population for the genetic algorithm:

- First, all chromosomes contain valuable genes instead of being randomly generated.
- Second, *GASHAP* accelerates genetic algorithm convergence by reducing the number of brain regions.
- Third, the *GASHAP* method uses fewer chromosomes for initialization, equal to the number of generated heatmaps.

In our previous study [2], we observed that the values of and with α = 0.025 and β = 0.975 resulted in improved performance compared to other sets of and values. Our experiments were carried out in two phases using the same values of and. In this study, we conducted two experiments after calculating Shapley values and using Equation 4 to determine the score of each brain region. The process of generating chromosomes for the experiment was illustrated in Fig 7, with a range of (0–30%), (30–50%), (50–70%), and (70–100%). The first experiment was conducted using the aforementioned range, in which the gene value of each brain region’s score was assigned as follows: (0–30%) = 0, (30–50%) = 1, (50–70%) = 2, and (70–100%) = 3. The morphological operation was applied based on each brain region’s gene value, as described in Table 4.

**Table 4:** Gene value assignment based on brain region importance and morphological operations (first experiment).

Range score	Gene value	The importance of the brain region	Morphological operation
(0-30)%	0	UR	The region is not considered and is omitted.
(30-50)%	1	IR	Erosion
(50-70)%	2	VIR	No morphological operation
(70-100)%	3	VVIR	Dilation

**Fig 8:**
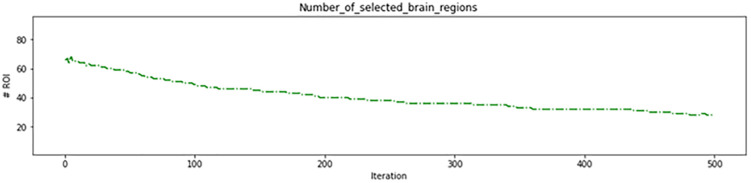
Number of brain regions of interest(ROI) found in genetic algorithm iterations in the first experiment.

**Fig 9:**
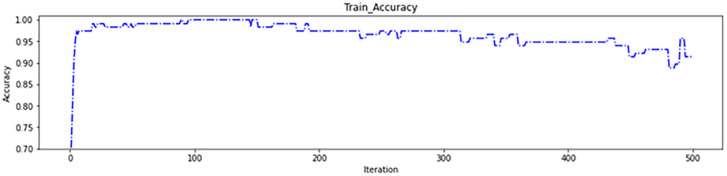
First experiment to train accuracy.

**Fig 10:**
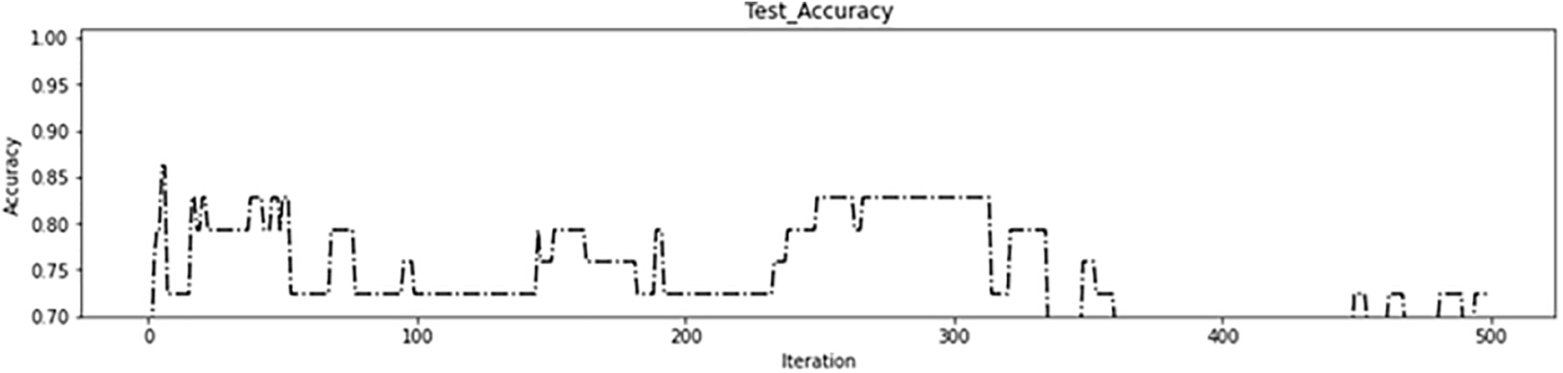
First experiment to test accuracy.

**Fig 11:**
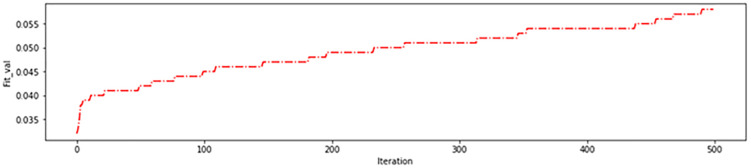
Fitness of the proposed approach in the first experiment.

As depicted in Fig 8, the genetic algorithm started with 62 regions in the initial iterations removing 34 ineffective regions before the algorithm began. This resulted in more than 95% accuracy on the training data (Fig 9) and more than 85% in the test data (Fig 10), which are acceptable levels of accuracy. These results indicate an accurate calculation of brain region scores and the correct identification of important regions. The fitness function value during genetic algorithm iterations is shown in Fig 11.

**Fig 12:**
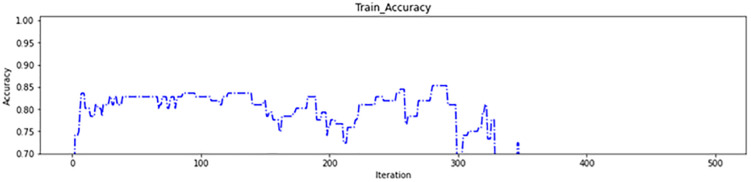
The second experiment train accuracy.

**Fig 13:**
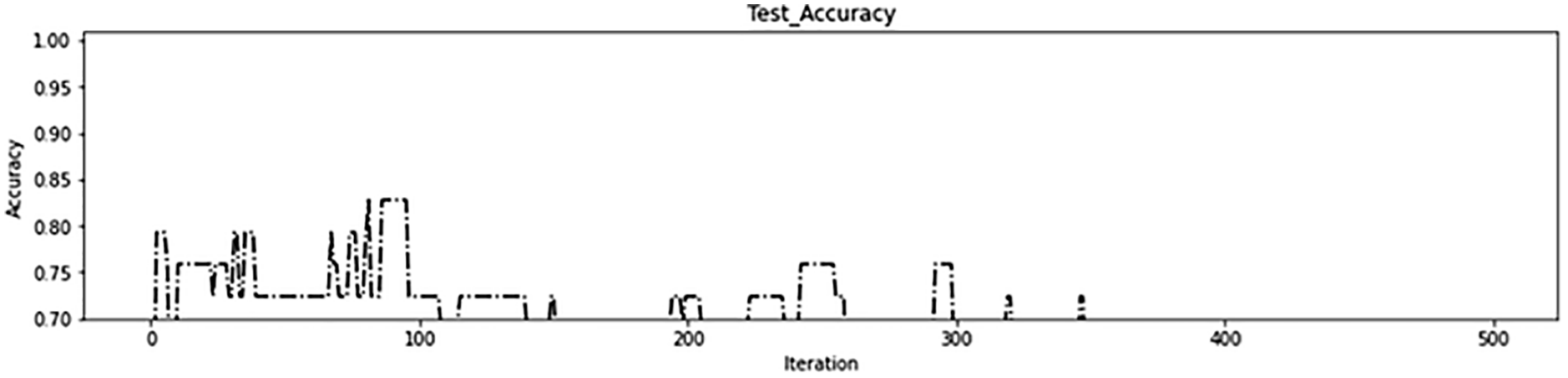
The second experiment test accuracy.

**Fig 14:**
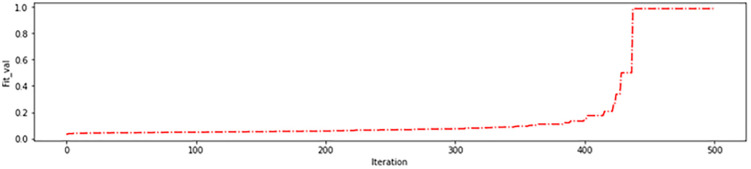
Fitness of the proposed approach in the second experiment.

**Fig 15:**
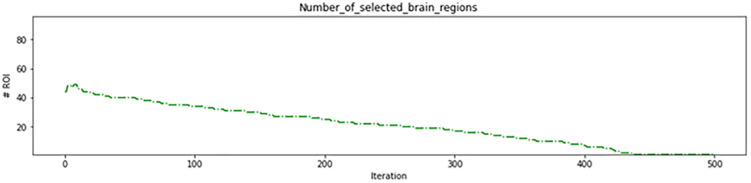
Number of ROI found in genetic algorithm iterations in the second experiment.

In the second experiment, a range of four intervals was employed: (0–40%), (40–60%), (60–80%), and (80–100%). This range was used to assign gene values to brain regions based on their scores. Specifically, the brain regions with scores within the range of (0–40%) were assigned a gene value of 0, those within (40–60%) were assigned a gene value of 1, those within (60–80%) were assigned a gene value of 2, and those within (80–100%) were assigned a gene value of 3. The morphological operation was applied to each brain region based on its assigned gene value, as detailed in Table 5. The genetic algorithm used in this experiment began with 50 brain regions in the first iteration, resulting in the removal of 46 ineffective regions. This approach achieved accuracy of 84% in both the training data (Fig 12) and test data (Fig 13) during initial iterations, which is considered acceptable. These results demonstrate the accuracy of the brain region score calculation and the correct identification of critical regions. The progression of the fitness function value during the genetic algorithm iterations is depicted in Fig 14, and the number of brain regions found in each iteration can be seen in Fig 15.

**Table 5:** Gene value assignment based on scoring ranges and morphological operations (second experiment).

range score	gene value	The importance of the brain region	Morphological operation
(0-40)%	0	UR	The region is not considered and is omitted.
(40-60)%	1	IR	Erosion
(60-80)%	2	VIR	No morphological operation
(80-100)%	3	VVIR	Dilation

In the conclusion of the first and second experiments, it was observed that as the number of removed regions increased, the model’s accuracy decreased gradually. This suggests that the remaining brain regions are more important and play a significant role in improving the model’s accuracy.

### 4.1. Discussion on brain regions discovered for Alzheimer’s disease

Table 6 presents the brain regions identified from the first and second experiments. The genetic algorithm was initially executed using the specified properties outlined in the first experiment. The resulting brain areas were then assigned to the set $R_{\text{1}}$. Following that, the genetic algorithm was re-executed using the specified properties outlined in the second experiment. The resulting brain regions were then assigned to the set $R_{\text{2}}$. $R_{\text{3}}$ is the set that can be defined as the set that contains elements that are common to both sets.

**Table 6:** Final brain regions identified by the GASHAP framework.

Region ID	Region name	Morphological operation(importance)	Gene	Reference
4	Left cingulate gyrus, posterior division	Dilation(VVIR)	3	[2,17,24,27,36]
9	Left frontal pole	No operation(VIR)	2	[7]
11	Left Inferior Frontal Gyrus, pars opercularis	Dilation(VVIR)	3	[13,36]
20	Left Lateral Occipital Cortex, superior division	Erosion(IR)	1	[2,8,12,23]
29	Left Parahippocampal Gyrus, anterior division	Dilation(VVIR)	3	[2,10,20,28,36]
41	Left Superior Temporal Gyrus, posterior division	No operation(VIR)	2	[36]
43	Left Supramarginal Gyrus, anterior division	No operation(VIR)	2	[36]
77	Right Parahippocampal Gyrus, anterior division	No operation(VIR)	2	[28,36]
86	Right Superior Frontal Gyrus	Dilation(VVIR)	3	[10,13]
89	Right Superior Temporal Gyrus, posterior division	Dilation(VVIR)	3	[10,36]
91	Right Supramarginal Gyrus, anterior division	Erosion(IR)	1	[36]

**Fig 16:**
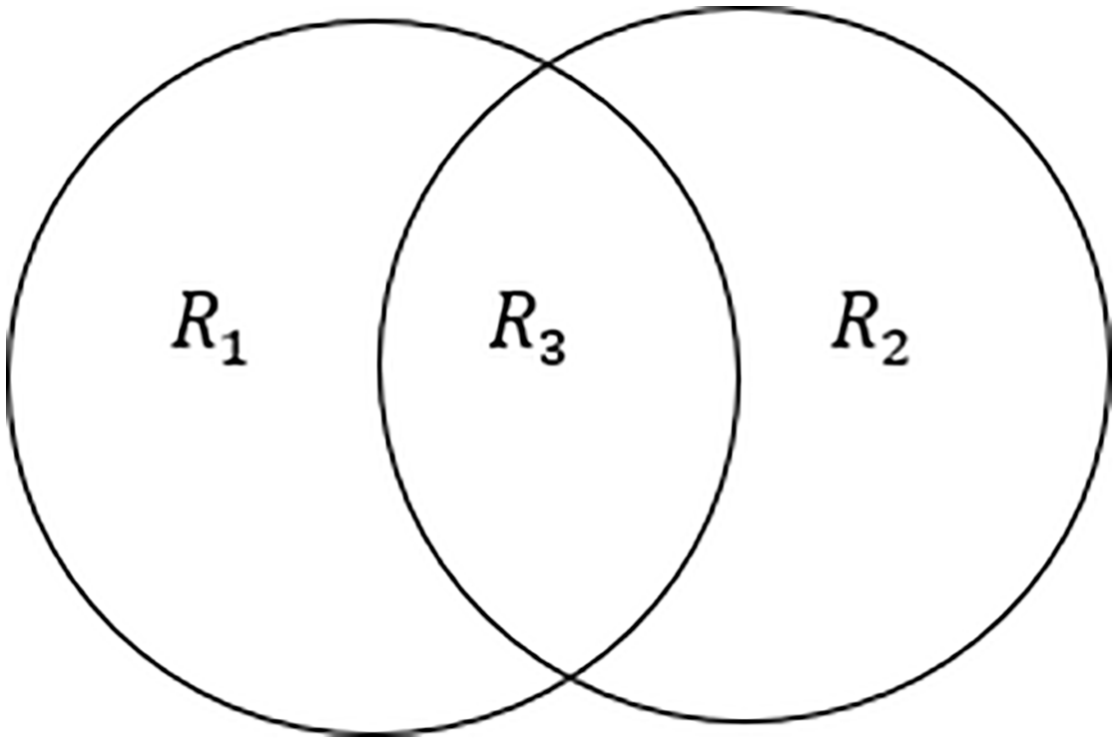
$R_{3}\;=\;R_{1}\;\cap \;R_{2}$.

The individuals comprising this are shown in Fig 16. The primary objective of the intersection of sets was to improve accuracy and reveal more exact findings. To achieve a thorough understanding of a heatmap, it is sometimes necessary to evaluate it at different scales. For example, through careful examination of the image at a reduced scale, one can perceive finer details, while analyzing the image at an enlarged scale offers a more comprehensive understanding of the sent information. To conduct a thorough analysis of our hypothesis, we employ the use of two experiments and their intersection. This approach allows for a comprehensive examination of the hypothesis across both experiments, yielding a more robust outcome.

The results of the *GASHAP* study have been previously published in several papers. Of particular relevance, a substantial portion of the identified brain regions demonstrated a gene value of 3, highlighting the robustness of this methodology. The study demonstrates high accuracy, as evidenced by its performance on training and test datasets, its convergence rate, and the number of regions identified in the initial stages. A comprehensive list of the identified brain regions is presented in Table 6, along with references to previous studies that have confirmed their validity. The brain regions identified in the study include:

- *Left cingulate gyrus, posterior division*
- *Left frontal pole*
- *Left Inferior Frontal Gyrus, pars opercularis*
- *Left Lateral Occipital Cortex, superior division*
- *Left Parahippocampal Gyrus, anterior division*
- *Left Superior Temporal Gyrus, posterior division*
- *Left Supramarginal Gyrus, anterior division*
- *Right Parahippocampal Gyrus, anterior division*
- *Right Superior Frontal Gyrus*
- *Right Superior Temporal Gyrus, posterior division*
- *Right Supramarginal Gyrus, anterior division*

**Fig 17:**
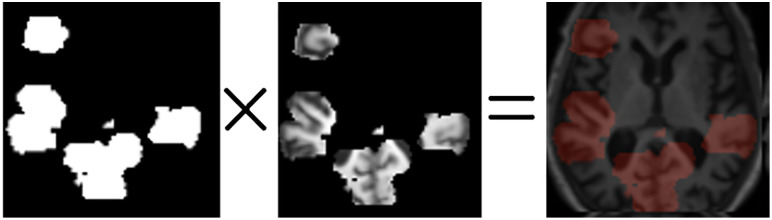
Discovered brain regions and final generated mask by GASHAP.

**Fig 18:**
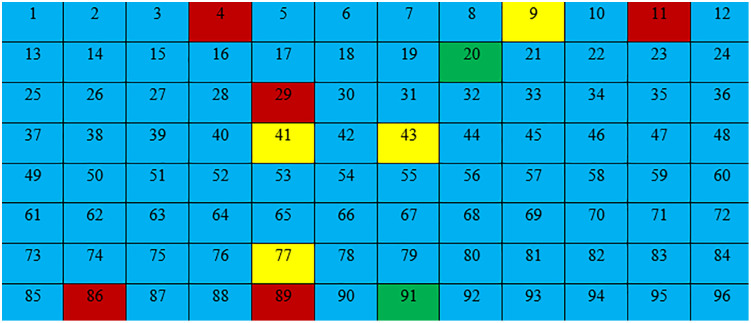
Final discovered chromosome.

**Fig 19:**
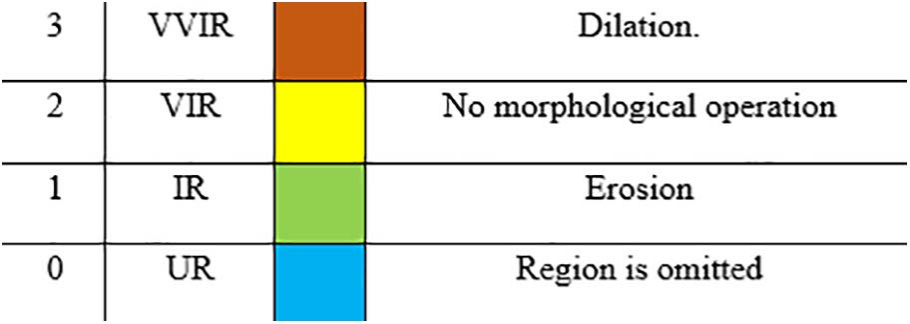
A guide for Fig 18 coloring.

**Fig 20:**
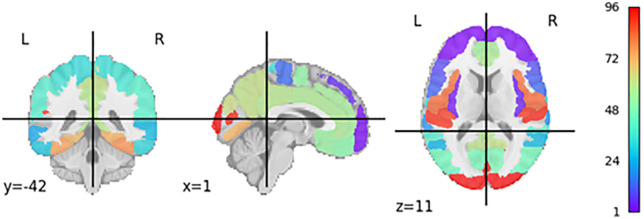
Harvard-Oxford cortical and subcortical structural atlas.

The final mask visualization generated by the study is shown in Fig 17. The corresponding chromosomes of the identified brain regions are displayed in Fig 18, with the coloring scheme explained in Fig 19. The details of the final results, along with relevant previous research, are presented in Table 6. The brain regions identified by the proposed GASHAP framework are summarized in **Table 7**, which lists the regions most relevant for Alzheimer’s disease classification (Fig 20). A comparison of the GASHAP framework with previous GA-based explainability studies is presented in **Table 8**.

**Table 7:** Extracted brain regions from GASHAP.

Region Id	Brain region name
4	Left Cingulate Gyrus, posterior division
9	Left Frontal Pole
11	Left Inferior Frontal Gyrus, pars opercularis
20	Left Lateral Occipital Cortex, superior division
29	Left Parahippocampal Gyrus, anterior division
41	Left Superior Temporal Gyrus, posterior division
43	Left Supramarginal Gyrus, anterior division
77	Right Parahippocampal Gyrus, anterior division
86	Right Superior Frontal Gyrus
89	Right Superior Temporal Gyrus, posterior division
91	Right Supramarginal Gyrus, anterior division

**Table 8:** Comparing GASHAP with previous studies.

Approach	Explanation of the approach	Creation of initial population	Advantage and disadvantages
[2]	This work establishes the initial population by assigning a random value from the set {0,1,2,3} to each gene, utilizing a predetermined probability. In this procedure, the length of each chromosome is 96. Following the execution of the genetic algorithm, the resulting output is the final mask that encompasses the significant brain areas.	random	**Advantages:** • One of the primary benefits of this approach is its ability to offer a novel means of elucidating the intricacies of the convolutional neural network • Introducing an innovative approach to elucidate the convolutional neural network **Disadvantages:** • The first population exhibits a substantial number of members. • The first population is generated in a random manner, resulting in the formation of chromosomes of low quality within the initial population.
[43]	In this paper, after employing explainable techniques based on backpropagation to train the neural network, a preliminary mask was generated for the purpose of utilizing the Occlusion Map. The process of finding a suitable mask for the occlusion map involves an iterative technique, which was accomplished using a genetic algorithm in this approach. Subsequently, the mask obtained in the preceding step was used as input for the genetic algorithm in order to obtain an accurate brain mask.	Presenting an intelligent method using heatmaps based on Backpropagation	**Advantages:** • One of the benefits of this approach is that it offers an intelligent method for generating the initial population. • This study aims to present the ultimate quality mask of brain regions. **Disadvantages:** • The first population exhibits a substantial number of members. • The utilization of averages in the computation of heatmaps may result in inaccuracies during the production of the final mask.
*GASHAP*	The GASHAP approach included the use of SHAP values and a score calculation formula to identify significant regions within each heatmap. Subsequently, a genetic algorithm was employed to derive the ultimate mask.	Creating the initial population with the help of SHAP values and the score calculation formula	• Using SHAP values to calculate scores of brain regions • Providing the final quality mask of brain areas. • Increasing the convergence speed of the genetic algorithm by removing unnecessary areas. • By avoiding the use of the average in the computation of the final mask, the objective is to minimize the error in the final results.

## 5. Discussion

In the previous part, we reported the brain regions identified in our study and conducted a more precise analysis by comparing them with existing studies in the field. This comparison aimed to determine whether our framework could successfully identify the relevant brain areas. Now we are going to go over the GASHAP framework, using the previous studies we made, and discuss why the GASHAP approach can be beneficial. The main difference between GASHAP and our other studies [2,43] lies in how the initial population and brain regions were selected. These two research publications investigate the use of genetic algorithms to identify an optimal brain mask for Alzheimer’s disease diagnosis. In essence, a computational approach is employed to identify the primary cerebral regions linked to Alzheimer’s disease, with the aim of using these regions within a convolutional neural network to diagnose the condition. In the study by [2], an approach for generating the initial population is employed, in which each gene is assigned a value from the set {0, 1, 2, 3} with a predetermined probability. The initial population consists of 200 individuals, with each individual associated with a distinct brain mask generated by the algorithm. The use of a genetic algorithm to identify an appropriate brain mask is also demonstrated in the aforementioned article [43]. Nevertheless, within the context of this study, it is important to note that each chromosome contains 96 genes, each corresponding to specific locations delineated in a brain atlas. Each gene in the genetic sequence might assume one of the discrete values {0, 1, 2, 3}. In this context, a value of 0 indicates that the corresponding region is not selected; a value of 1 indicates that it is selected; and values of 2 and 3 indicate selection of erosion operators and expansion, respectively. The application is carried out in the designated area, if appropriate. By multiplying each brain atlas by the voxel mask and dividing the result by the total number of voxels in that particular individual atlas, one can determine the starting population. The initial chromosome assigns a value of 1 to the genes associated with the top N brain areas exhibiting the highest ratio. The first population is generated with heatmaps based on backscatter. Table 6 discusses the GASHAP approach and its alignment with previous studies, providing details.

The precision of the models used, and the number of discovered regions can be compared between the outcomes of this study and those of other scholarly publications.

The *GASHAP* experiment involved sharing areas generated from both the original and subsequent tests, resulting in a total of 11 areas.

These regions have been previously designated as significant in scientific publications of high academic standing.

The justification for selecting a smaller, more accurate set of areas in this study is the deliberate reduction of the initial population and the development of precise chromosomes before commencing the genetic algorithm. Supplementary data can be found in Table 9.

**Table 9:** Comparison of the proposed method in terms of the number of brain regions obtained and the accuracy of the model.

Reference	Train accuracy		Test accuracy	Founded brain regions
*GASHAP*	First experiment	96%	85%	11
	Second experiment	84%	84%	
[2]	96%		85%	41
[43]	Between 75% to 94% accuracy		-------	18 to 41 brain regions
[29]	96%		96%	Not specified
[27]	97.8-99%		98.3-99%	Not applicable (clinical data)

## 6. Conclusion

We have presented a novel XAI method, *GASHAP,* for diagnosing Alzheimer’s disease. Firstly, we classified the brain MRI images in the ADNI dataset into two predefined groups using CNN. Then, we used the SHAP library’s deep explanation to generate heatmaps that highlight the key voxels in each MRI image, thereby obtaining a heatmap for each image that identifies the most and least important voxels. To increase explainability, we translated these results into brain regions using the Harvard University Atlas and selected significant regions using a genetic algorithm with a new chromosome-encoding scheme to initialize the population. The outcome of this study provides a trustworthy list of critical brain regions that are crucial for an accurate diagnosis of Alzheimer’s disease. The present study opens avenues for future investigations, including:

1. Exploration of different brain atlases to represent the results in alternative formats.
2. Expansion of the dataset and clustering of heatmaps, followed by the application of a genetic algorithm to each cluster to identify distinct patterns of Alzheimer’s disease.
3. Using different XAI methods to be combined with genetic algorithms to distinguish the differences in the results.
